# COVID-19 home remedies and myths becoming a hazardous health infodemic?

**DOI:** 10.4102/jamba.v13i1.1115

**Published:** 2021-09-30

**Authors:** Olivia Kunguma

**Affiliations:** 1 Disaster Management Training and Education Centre for Africa, Faculty of Natural and Agricultural Sciences, University of the Free State, Bloemfontein, South Africa

**Keywords:** COVID-19, infodemics, public health, home remedies and myths, Disaster Management Act

## Abstract

Coronavirus disease 2019 (COVID-19) brought on several social, economic, political, and environmental challenges. What was mostly questioned was the efficacy of the *Disaster Management Act* 57 of 2002 (As Amended 16 of 2015) (DMA), which was used to declare COVID-19 a disaster. The concern was whether the DMA is able to deal with pandemics when its focus is mostly on climate-related disasters. Most public health emergencies experience the spread of overwhelming information, some of which may be true and others may be false information. This article discusses the home remedies and myths related to COVID-19, that could impede pandemic response efforts. Subsequently, this study raises a question regarding the effectiveness of DMA to deal with such types of compounding risks. In doing so, this research is exploratory where the DMA and the media articles on COVID-19 home remedies and myths are systematically reviewed. Coronavirus disease 2019 home remedies and myths were found to be hazardous and the DMA was found unprepared to deal with such types of compounding risks. ‘Infodemic management’ needs to be considered in the DMA in order to prepare for effective disaster response.

## Introduction

The entire world is at risk of coronavirus disease 2019 (COVID-19) with millions of lives lost, millions infected, and livelihoods affected. Governments’ efforts to prevent and mitigate the effects of the pandemic are somehow compromised. Mostly, citizen journalists and even some government officials disseminate information that impedes the governments’ efforts to fight the pandemic. The immense distribution of COVID-19 information, most of which is misinformation and disinformation, has unveiled to the governments the need and urgency to prevent and mitigate the spread of fake news. Because COVID-19 in South Africa was declared a disaster through the guidance of the *Disaster Management Act* 57 of 2002 (As Amended 16 of 2015) (DMA) (Kunguma, Ncube & Mokhele 2021; Republic of South Africa 2002), this study poses a question on whether this legislation anticipated the possibilities of fighting an infodemic alongside a disaster.

The issues at hand are the problems that emerge with a public health emergency, in this case, COVID-19. The emerging problems are the overwhelming spread of information, some of it true and some of it fake. All of this occurs, amidst the efforts to mitigate and prevent the health issue. Consequently, this situation impedes the pandemic response efforts, in most cases exacerbating the health emergency (Gallotti et al. 2020). In a situation like this, policies and legislation must be in place to effectively manage complex situations like these.

Therefore, the objective of this research is to review the DMA in the context of its approach towards promoting science-based information to disseminate during a disaster thereby preventing ‘infodemics’. This article examines the concept of ‘infodemic’ and recalls some of the COVID-19 myths and home remedies. Furthermore, COVID-19 infodemic risk reduction strategies are discussed, followed by the discussion of the ‘infamous’ South African DMA and a conclusion.

## Methodology

This research conducted a qualitative exploratory study in South Africa, expressing opinions on the COVID-19 home remedies and myths that were compounding risks. The study also set to investigate the South Africans DMA in its aptness to deal with such types of compounding risks. Secondary data was collected through the purposeful review of media articles on COVID-19 myths and home remedies. The DMA was systematically reviewed searching for the following keywords in its database (Smith et al. 2011): *Information, Infodemics, Public Health Information, Media, Communication, Fake News, Social Media, and Technology*. These words were relevant to understand if the DMA mandates the management of ‘infodemics’.

### Understanding the word ‘infodemic’

Whilst the word ‘infodemic’ may seem like a new word, it is not. The word was coined in 2003 as a blend of the words ‘information’ and ‘epidemic’ during the Severe Acute Respiratory Syndrome (SARS) outbreak. During this time, the world experienced two viral epidemics, the SARS and the spread of information about the virus (Merriam-Webster 2021). The word ‘infodemic’ means the viral spread of overwhelming information and misinformation about a health emergency (United Nations 2020). As a result, confusion, distrust, and panic developed amongst the affected public (Department of Global Communications 2020). The worst end result of infodemics is that effective public health response is adversely affected and undermined (World Health Organisation 2020). The declaration of COVID-19 as a global pandemic in 2020 revived the word infodemics, which exposed the global population to many myths and home remedies related to it. With this regard, it is important to understand the meaning of the word ‘myth’, which is defined in the Merriam-Webster Dictionary as a widely held false belief or idea (Merriam-Webster 2021). The myths and remedies related to COVID-19 were disseminated amongst the public at the same speed as the spread of the virus. Most of the myths and remedies have done more harm than good.

### COVID-19 myths and home remedies

This section unpacks some of the myths and home remedies that brought on controversy amongst the global population. Table 1 illustrates the myths and home remedies, including a brief description of each.

**TABLE 1:** COVID-19 myths and home remedies.

Number	Variable
**Myths**	
1.	Black and Asian people globally are more at risk from contracting COVID-19 because of deficiencies in Vitamin D.
2.	Vaccine will cause infertility
3	No need to wear a mask after vaccination
**Home remedies**	
1.	Vitamin D prevents and treats COVID-19 (e.g. spend time in sunlight)
2.	Alkaline foods eliminate the virus
3.	Inhaling steam with herbs would paralyse the virus.

*Source*: Broughton and Jarvis (2020), Hancocks (2020), Reuters (2020), Sample (2020), The Lancet Diabetes and Endocrinology (2021), Times of India (2021), Wen (2021)

Note: Please see the full reference list of the article. Arifin, S., Wicaksono, S.S. & Sumarto, S., 2021, ‘Coronavirus 2019 home remedies and myths becoming a hazardous health infodemic?’, *Jàmbá: Journal of Disaster Risk Studies* 13(1), a1115. https://doi.org/10.4102/jamba.v13i1.1115, for more information.

COVID-19, coronavirus disease 2019.

With reference to #1 in the Table, by following the myth about black and Asian people, a belief that Vitamin D prevents and treats COVID-19 emerged (Hancocks 2020). On the contrary, this myth is argued by several authors that it is more of the socio-economic status of the black and Asian people than their genetics (Broughton & Jarvis 2020; Sample 2020). Then the Vitamin D remedy was also found to lack evidence and is still under investigation (The Lancet Diabetes & Endocrinology 2021). Another remedy suggested was the consumption of alkaline foods to eliminate the virus (Times of India.com 2021). In an article by an International News Agency (Reuters 2020), social network users shared the content that inhaling steam with herbs (such as eucalyptus oil, garlic, ginger, tea tree and so on) would paralyse the virus. World Health Organization dismissed these remedies, arguing that COVID-19 has nothing to do with the stomach (World Health Organisation 2021a). In fact, too much lemon builds up acid in the stomach, and steaming can even cause injuries (Eyewitness News 2021). Supplements and herbs like eucalyptus, ginger, Vitamin C, Zinc and many other indigenous herbs have also become common household favourites following the outbreak of the COVID-19 pandemic (Kgobotlo 2020). Many people believe that these herbs and vitamins mentioned above prevent the COVID-19 infection. Eventually, when COVID-19 vaccines were developed and released, there was a wave of misinformation about the vaccines. For example, there was a myth that the vaccine will cause infertility because of the spike proteins in COVID-19 and the Syncytin-1 protein that helps with placenta development. Another myth is that there is no need to wear a mask after getting vaccinated (Wen 2021). These rumours about the vaccines caused hesitancy amongst the public to get vaccinated (Kelen & Maragakis 2021). The above mentioned remedies and myths are just a few, and citizen journalists disseminated them on platforms like WhatsApp, Twitter, Facebook, and offline.

The downside of misinformation is that most people have relied heavily on and trusted the information so much that they would not seek medical attention. They would treat themselves at home with the unofficially recommended remedies. In some cases, some infected people who could have been treated with official medicines lost their lives whilst trying to self-medicate with unofficial remedies (Park 2020; Wright 2020). A significant number of people have lost their lives, not from the COVID-19 infection but from self-medicating with chemicals like Chloroquine Phosphate (Upham 2020).

### COVID-19 infodemic risk reduction strategies so far

The escalation in infodemics prompted institutions like the World Health Organization into initiating events and programmes to fight the spread of fake news. The initiatives were the third Virtual Global WHO Infodemic Management Conference, where stakeholders came together to affirm their commitment to mitigate the impacts of the COVID-19 infodemic. Another initiative was the Africa Infodemic Response Alliance to detect, disrupt and counter public health misinformation in Africa (United Nations 2020). Furthermore, WHO built a platform that they called Early Artificial-Intelligence-supported Response with Social Listening (EARS). This platform pulls trending topics from major platforms like Facebook, Twitter, and many others. Officials can identify people’s intentions, that is, whether they are speculations or questions, and as a result, the officials can address the speculations mitigating possible chances of conspiracy theories (World Health Organization 2021). In South Africa, the President took note of the fact that there was a possibility of misinformation to reach an ignorant population and that the spread of fake news needed to be regulated. To combat infodemics, South Africa established a National Coronavirus Command Council (NCCC) with the objective to advise the government and create awareness of the virus. Emanating from the NCCC, the government declared the act of spreading fake news a criminal offence punishable by fine or jail. A Digital Complaints Committee was established to handle misinformation reports that can be reported through a hotline or WhatsApp line (Jimoh et al. 2020).

Fighting these myths and home remedies is sometimes difficult because even some politicians are the culprits (Park 2020). In South Africa, it was the African National Congress Youth League that launched an unofficial ‘herb steaming campaign’ and the offenders were later arrested (Tandwa 2021). In Tanzania, the late President Magufuli believed that COVID-19 cases were being artificially exaggerated and he trusted herbs from Madagascar (Paget & Kwayu 2020). In China, government health officials strongly recommended traditional Chinese medicines. China also exported the recommended medicines to other countries like Italy (Cyranoski 2020).

### South African *Disaster Management Act* and infodemics prevention

So far, ‘infodemics’ have been associated with the dissemination of public health information. Moreover, because the DMA was promulgated as a result of a severe increase in climate-induced disasters, it is no surprise that the legislation was never prepared to deal with the compounding ‘infodemic hazard’. According to the DMA, the definition of a disaster is stated as a natural or human-induced occurrence that causes or threatens to cause disease and not a disease that threatens to cause a disaster. In this case, COVID-19 seems to have challenged this definition.

In the DMA database, a full-text screening was carried out, searching for the following keywords: ‘Information (34 mentions), Infodemics (0 mentions), public health information (0 mentions), Media (0 mentions), Communication (11 mentions), Fake News (0 mentions), Social Media (0 mentions) and Technology (0 mentions)’. These words were relevant to what was under investigation. Deriving from the systematic review results, it is evident that the DMA does not prescribe the handling of overwhelming information, and misinformation before or during a disaster. The most significant prescription in the DMA about disaster information is that the national centre must act as a repository and conduit for disaster information. Therefore, the national centre’s mandate is to establish an electronic database that collects, process and disseminate information to disaster vulnerable communities. The database is supposed to include information that causes or aggravate disasters and the various means to reduce the risks. The DMA also gives the different spheres of disaster management the power to request organs of state and any other person in possession of information to release it.

The national disaster management framework, a prescription of the DMA which was developed to guide disaster risk management and response, unpacks in detail the management and distribution of information. It prescribes the staffing of information scientists in the disaster management centres, establishment of a central 24-h communication centre, and managing the vulnerability, risk assessment and dissemination of weather-related information, and so on. A review of the DMA and the framework revealed that they both support and guide the management, maintenance and dissemination of information amongst interest stakeholder groups for the purpose of disaster risk reduction and response. Whilst all the information and communication mechanisms exist in the DMA and framework to guide the development of COVID-19 infodemic management strategies, the reference to public health information management is missing in these statutes. The statutes fail to mention the management of public perceptions, misinformation and disinformation that might impede their disaster response efforts.

## Conclusion

It is evident that the home remedies and myths about COVID-19 have become a hazardous health ‘infodemics’ that impedes the pandemic response efforts. And whilst various measures were put in place to respond to the negative effects of ‘infodemics’, the DMA which was used to declare COVID-19 a disaster, is silent on addressing this issue. The DMA is, however, strong on its prescription of the establishment of information and communication systems, gathering of information, formation of communication links and the dissemination of information. But these prescriptions are insufficient and not clear on how disaster managers can handle the overwhelming information and misinformation that affects their response efforts. Because the disaster management legislation clearly states that the disaster management centre must act as a repository and conduit for information, then all the data about COVID-19 information, misinformation and disinformation should be with the centre. The availability of such data makes it easier for the centre to develop infodemic prevention and mitigation strategies. A significant subject for consideration in the future review of the DMA is the inclusion of ‘Infodemic Management’. Infodemic management is the application of evidence-based interventions to provide citizens with localised, factual and understandable information that will enable them to acquire positive health-seeking behaviour (World Health Organization 2020). Realising that ‘infodemics’ are a compounding hazard, the disaster management policymakers need to seriously consider this issue. Disaster management policymakers need to consider the fact that the digital world has increased the risk of infodemics. Therefore, a strong emphasis should be placed on the need for a DMA that supports the management of disaster risks through technology, social media, evidence-based research, perception management and community participation in infodemic management (more of community policing).
