# Erratum: COVID-19 home remedies and myths becoming a hazardous health infodemic?

**DOI:** 10.4102/jamba.v13i1.1229

**Published:** 2021-12-13

**Authors:** Olivia Kunguma

**Affiliations:** 1 Disaster Management Training and Education Centre for Africa, Faculty of Natural and Agricultural Sciences, University of the Free State, Bloemfontein, South Africa

In the published article, Arifin, S., Wicaksono, S.S. & Sumarto, S., 2021, ‘COVID-19 home remedies and myths becoming a hazardous health infodemic?’, *Jàmbá: Journal of Disaster Risk Studies* 13(1), a1115. https://doi.org/10.4102/jamba.v13i1.1115, the article citation was given incorrectly. The citation was given as ‘Arifin, S., Wicaksono, S.S. & Sumarto, S., 2021, ‘COVID-19 home remedies and myths becoming a hazardous health infodemic?’, *Jàmbá: Journal of Disaster Risk Studies* 13(1), a1115. https://doi.org/10.4102/jamba.v13i1.1115’. The correct citation should be ‘Kunguma, O., 2021, ‘COVID-19 home remedies and myths becoming a hazardous health infodemic?’, *Jàmbá: Journal of Disaster Risk Studies* 13(1), a1115. https://doi.org/10.4102/jamba.v13i1.1115.

The publisher apologises for this error. The correction does not change the study’s findings of significance or overall interpretation of the study’s results or the scientific conclusions of the article in any way.

